# Combined CMR and catheterization for pre-Fontan evaluation: comparing metrics and clinical correlations

**DOI:** 10.1016/j.jocmr.2026.102723

**Published:** 2026-04-06

**Authors:** Ashish Shrivastava, Russel Hirsch, Shabana Shahanavaz, Cara E. Morin, Todd Jenkins, Zhiqian Gao, Sean M. Lang

**Affiliations:** a The Heart Institute, Cincinnati Children’s Hospital Medical Center, Cincinnati, Ohio, USA; b Department of Pediatrics, University of Cincinnati College of Medicine, Cincinnati, Ohio, USA; c Department of Radiology, Cincinnati Children's Hospital and University of Cincinnati College of Medicine, Cincinnati, Ohio, USA; d Heart Institute Research Core, Cincinnati Children’s Hospital Medical Center, Cincinnati, Ohio, USA; e Division of Biostatistics & Epidemiology, Department of Pediatrics, University of Cincinnati College of Medicine, Cincinnati, Ohio, USA

**Keywords:** Cardiac magnetic resonance, Single ventricle, Cath, Ejection fraction, Aortopulmonary collateral

## Abstract

**Background:**

Cardiovascular magnetic resonance imaging (CMR) is increasingly used in combination with cardiac catheterization (Cath) for the pre-Fontan evaluation of children with single-ventricle physiology. The aim of this study was to describe our institutional experience of this combined assessment.

**Methods:**

We conducted a retrospective, single-center study of 57 single-ventricle patients who underwent CMR and Cath under the same anesthesia prior to Fontan completion. CMR and Cath-based pulmonary and systemic blood flow ratio (Qp/Qs) and aortopulmonary collateral (APC) assessments were compared. In addition, we analyzed the association between significant APC burden (>30% of aortic flow) and preoperative hemodynamics, as well as postoperative outcomes, including chest tube duration and hospital length of stay.

**Results:**

CMR consistently yielded higher Qp/Qs values compared to Cath, with a mean underestimation of 0.41 by the latter. This difference was strongly correlated with CMR-derived APC burden (p<0.0001). In comparison, Cath-based qualitative assessment of the APC had a poor correlation with the CMR APC burden and Qp/Qs. The CMR APC burden was also associated with increased indexed ventricular volumes and lower ejection fraction.

**Conclusion:**

Combined CMR and Cath provide a comprehensive pre-Fontan assessment, with CMR offering superior quantification of aortopulmonary collateral burden and demonstrating larger Qp/Qs than calculated by Cath methods. These findings support the continued use of integrated imaging modalities for individualized care.

## Graphical abstract

**Figure:**
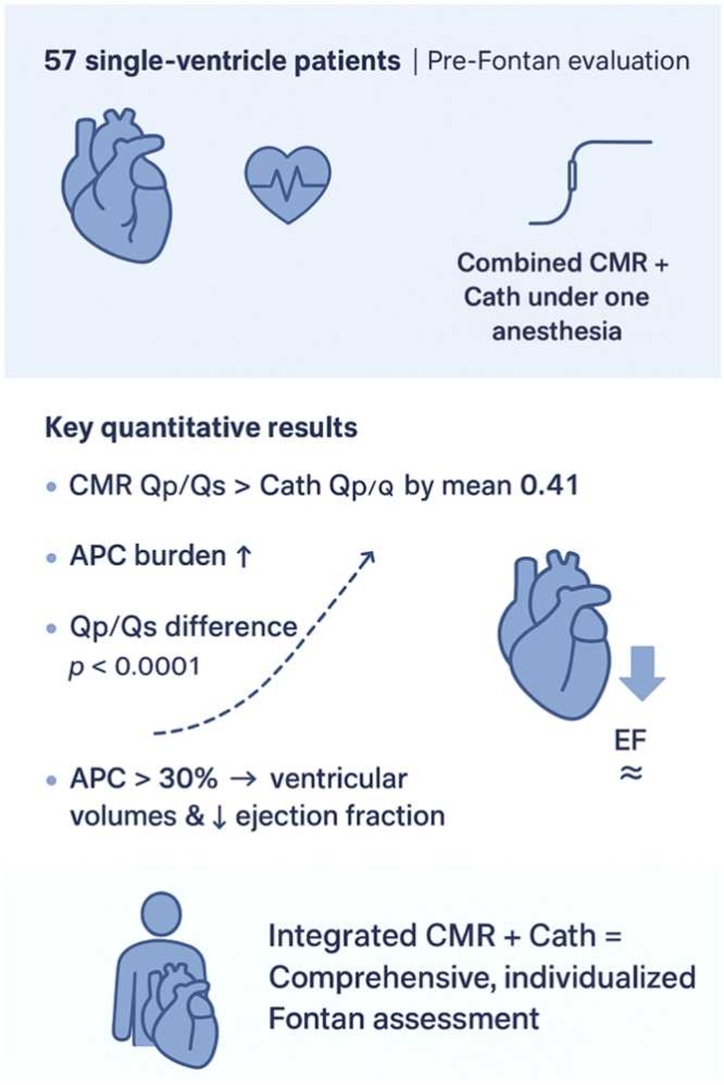

## 1 Introduction

Over the past five decades, advancements in surgical and medical management have markedly improved survival for patients with single-ventricle (SV) physiology. However, long-term morbidity and mortality remain substantial, with less than half of patients surviving over 30 years of age [1]. A comprehensive preoperative assessment is critical for optimizing outcomes following Fontan palliation, the final stage in SV surgical palliation. Cardiac catheterization (Cath) has been accepted as the standard for pre-Fontan assessment, allowing for direct pressure measurements and possible pre-surgical interventions [2]. Cardiovascular magnetic resonance (CMR) has become an invaluable imaging tool over the last several decades, allowing for a noninvasive modality to visualize complex anatomy as well as provide volumetric, functional, and flow data without geometric assumptions [3]. CMR is the standard of care for long-term surveillance of SV physiology because of its ability to predict death or transplantation [4], [5], [6]. In the pre-Fontan population, CMR-derived aortopulmonary collateral (APC) flow correlates with prolonged chest tube output and surgical length of stay at the time of Fontan surgery [7], [8], [9], [10]. As demonstrated by Eilers et al., combined CMR and Cath pre-Fontan assessment decreases Cath procedure and fluoroscopy time and decreases unnecessary collateral coiling [11]. This has prompted our institution to standardly perform CMR prior to pre-Fontan Cath. The aim of this study was to compare our CMR and Cath assessments of APC and Qp/Qs ratio and the associations with preoperative and postoperative metrics.

## 2 Methodology

### 2.1 Study design

We conducted a single-center retrospective study on single-ventricle patients at the Cincinnati Children’s Hospital that was approved by the Institutional Review Board. Patients who underwent pre-Fontan evaluation with CMR along with cardiac Cath done under the same general anesthesia from January 1, 2018 to December 31, 2024 were included. Cath data was collected using the Improving Pediatric and Adult Congenital Treatments (IMPACT) data registry. Hospital and surgical outcomes data were collected from the electronic medical record.

### 2.2 Exclusion criteria

Patients with surgical pathways other than Fontan palliation were excluded. From the CMR perspective, we excluded patients with poor image quality, resulting in the inability to perform the necessary cardiac measurements. Patients with CMR and Cath, if not performed under the same general anesthesia, were also excluded.

### 2.3 CMR protocol

All CMR studies were performed under general anesthesia with similar conditions to the subsequent cardiac catheterization, such as room air when possible. Studies were performed using a 1.5T scanner (Ingenia, Philips Healthcare, Best, the Netherlands). Volumetric and functional assessment was made using a standard cine balanced steady-state free precession (bSSFP) short-axis stack from the atrioventricular valve annulus to the apex. Typical parameters for bSSFP images were field of view 180 × 180 mm, matrix size 120 × 80 mm, slice thickness of 6 mm without gap, pixel resolution 1.8 mm × 1.8 mm, TR/TE 2.7/1.3, 30 phases per R-R interval, and parallel imaging factor 2. Through-plane phase contrast sequences were performed of the superior vena cava (SVC), inferior vena cava (alternatively, the descending aorta at the diaphragm was used as a surrogate), right and left pulmonary veins, right and left pulmonary arteries, and neo or native aortic valve. Typical parameters for phase contrast images were a slice thickness of 6 mm, pixel resolution of 1.1 × 1.1 mm, TR/TE of 5.4/3.4, 30 phases per R-R interval, and parallel imaging factor 2. Our typical protocol involves dynamic contrast angiography as well as a 3D bSSFP whole heart sequence for planning of phase contrast sequences as well as vessel measurements. Volumetric and flow data were measured using CVI42 (Circle Imaging, Calgary, Alberta, Canada) post-processing software. Clinical studies are reviewed by both attending-level radiologists and cardiologists for accuracy. Aortopulmonary collateral burden was measured using previously reported methods [9], [12]. In brief, the average difference between pulmonary venous return and branch pulmonary artery flow and aorta and systemic venous flow was averaged. The percentage of collateral burden was obtained with aortic/neo-aortic valve flow chosen as the denominator. A higher APC burden was defined as APC >30%. Pulmonary artery measurements were obtained using post-contrast respiratory navigated, electrocardiogram-gated, 3D angiography. CMR Qp/Qs were measured using the total pulmonary vein flow as Qp and SVC and descending aorta flow as Qs.

### 2.4 Cath protocol

Cardiac Cath was performed under the general anesthesia initiated for the CMR. Hemodynamic measurements were obtained, when possible, on room air. Pressure and oxygen saturations were measured in the SVC, pulmonary arteries, inferior vena cava, atria, single ventricle, ascending aorta, and descending aorta. Hemodynamic data was calculated using the Fick principle, including cardiac index, the ratio of pulmonary to systemic blood flow, transpulmonary gradient, and pulmonary vascular resistance. APCs were evaluated through direct angiographic visualization during cardiac Cath. The decision to coil the APC was guided by angiographic findings, hemodynamic data, and APC measurements obtained from CMR.

A more objective-based Cath collateral grading system was made to address the setbacks of Cath-based subjective grading. Our approach incorporates the presence of collaterals originating from the internal mammary artery, as well as the saturation difference between the pulmonary arteries and the SVC. This methodology results in an 8-point grading scale for APCs (Table 1).

**Table 1:** Cath-based APC grading

Grading	Saturation difference (SVC-PA) %	Obvious IMA collaterals
1	<1	No
2	<1	Yes
3	1–5	No
4	1–5	Yes
5	6–10	No
6	6–10	Yes
7	>10	No
8	>10	Yes

*SVC* superior vena cava, *PA* pulmonary artery, *IMA* internal mammary artery

### 2.5 Data analysis

The continuous variables were expressed as means with median (interquartile range). Categorical variables were expressed as frequencies with percentages. The Qp/Qs measurements using CMR and cardiac Cath were compared with Bland-Altman analysis. General linear regression was performed to evaluate the association between Qp/Qs and secondary outcomes, including chest tube duration and length of stay. The Kruskal-Wallis test was used to assess whether there are correlations with continuous variables and a collateral burden of >30%. The threshold of 30% was chosen as it has been used in other publications as a criterion for coiling APC [11]. General linear regression was used to determine whether a collateral burden of >30% was associated with secondary outcome parameters, including chest tube duration and length of stay.

## 3 Results

A total of 57 patients met the inclusion criteria and underwent CMR and cardiac Cath under the same anesthesia for pre-Fontan evaluation. Of these, 47 proceeded to Fontan completion surgery, while 9 patients were awaiting Fontan surgery and 1 patient died prior to surgery. The cohort had a median age of 3.77 years and a median weight of 14.4 kg at the time of CMR/Cath. There was a predominance of males (67.7%). The majority of the cohort had right ventricular dominance (80.7%), with the most common diagnosis being hypoplastic left heart syndrome (HLHS) (59.6%). Heterotaxy was identified in 19%. Collateral burden exceeded 30% in 52.6% of patients with an APC range of 9% to 56%. No significant differences were seen in the demographic data based on the collateral burden >30%.

CMR data are provided in Table 2. Patients with higher APC (>30%) show several differences, including a higher Qp/Qs (1.2 vs 0.9; p<0.0001), increased indexed end-diastolic volumes (EDVi: 132 mL/m² vs 112 mL/m²; p = 0.011), greater indexed end-systolic volumes (ESVi: 71.5 mL/m² vs. 56 mL/m², p = 0.001), and smaller left pulmonary artery diameters (p = 0.029) compared to patients with lower (<30%) collateral burden. APC collateral burden is inversely correlated with dominant ventricular function (r = 0.3, p = 0.025) (Fig. 4).

**Table 2:** Patient CMR variables

Parameters	N	Overall	Collateral burden >30%		
		Overall (N = 57)	No (N = 27)	Yes (N = 30)	p-value
Heart rate (Beats/min)	57	106 (96, 114)	103 (96, 112)	109 (95, 118)	0.36
Ventricular EF (%)	57	48 (45, 51)	50 (47, 53)	48 (44.8, 51)	0.11
Ventricular end-diastolic volume, indexed (mL/m^2^)	57	121 (105, 150)	112 (99, 126)	132 (113, 157)	0.011
Ventricular end-systolic volume, indexed (mL/m^2^)	57	65 (54, 75)	56 (47, 69)	71.5 (61, 86.5)	0.001
Dominant Myocardial mass, indexed (g/m^2^)	57	43 (31.5, 52)	43 (31, 48)	42.5 (31.5, 58.3)	0.35
Collateral burden (%)	57	30 (23.5, 35)	23 (19, 26)	34.5 (31.8, 42)	<.0001
AVVR	57				0.7
trivial/mild		50 (87.7%)	23 (85.2%)	27 (90.0%)	
moderate		7 (12.3%)	4 (14.8%)	3 (10.0%)	
RPA average diameter (mm)	56	8 (7, 9.19)	8.25 (7, 9.25)	7.5 (6.88, 8.63)	0.18
LPA average diameter (mm)	55	6 (5, 8)	7 (5.5, 9)	5.5 (4.63, 7.25)	0.029
RPA percentage (%)	57	65 (54, 70)	60 (50, 69)	69 (55.5, 71.5)	0.09
LPA percentage (%)	57	35 (30, 46)	40 (31, 50)	31 (28.5, 44.5)	0.09
CMR Qp/Qs ratio	56	1.02 (0.9, 1.24)	0.9 (0.8, 1)	1.2 (1.02, 1.45)	<.0001

*AVVR* atrioventricular valve regurgitation, *EF* ejection fraction, *RPA* right pulmonary artery, *LPA* left right pulmonary artery, *CMR* cardiovascular magnetic resonance

Cath data are provided in Table 3. The median Qp/Qs ratio measured by Cath was 0.6 and not significantly different between the higher and lower APC groups as defined by CMR. Patients with higher APC burden demonstrated significantly increased indexed ventricular end-diastolic pressure (EDP: 8 mmHg vs. 7 mmHg, p = 0.046). No other significant differences in Cath variables were observed between groups with higher and lower APC burden. Most patients in our cohort had Cath collateral grading scores of 2 (51.8%) or 4 (33.9%). All the patients with oxygen step up from the SVC to PAs had notable IMA collaterals, resulting in no scores of 3, 5, or 7. Cath collateral scores were not statistically different between the higher and lower APC burden groups. Aside from collateral coiling, 7 of 57 patients (12.3%) underwent balloon or stent angioplasty of the branch pulmonary arteries, and only 1 of 57 patient (1.8%) required stent angioplasty of the aorta.

**Table 3:** Patient cardiac cath variables

Parameters		Overall	Collateral burden >30%		
	N	Overall (N = 57)	No (N = 27)	Yes (N = 30)	p-value
Qp/Qs ratio	57	0.6 (0.54, 0.68)	0.6 (0.52, 0.64)	0.63 (0.56, 0.76)	0.25
Cardiac Index (mL/min/m^2^)	57	5 (4.23, 6.12)	4.99 (4.22, 5.69)	5.24 (4.28, 6.24)	0.35
Ventricular end-diastolic pressure, indexed (mL/m^2^)	57	8 (7, 9)	7 (6, 9)	8 (7.75, 9)	0.046
Ventricular SBP (mmHg)	57	82 (79, 88)	82 (79, 88)	82 (78.5, 90.5)	0.67
Cath Collateral Grading	56				0.39
1		6 (10.7%)	3 (11.1%)	3 (10.3%)	
2		29 (51.8%)	17 (63.0%)	12 (41.4%)	
4		19 (33.9%)	7 (25.9%)	12 (41.4%)	
6		1 (1.8%)	0 (0.0%)	1 (3.4%)	
8		1 (1.8%)	0 (0.0%)	1 (3.4%)	
Procedure type	57				0.28
None		9 (15.8%)	6 (22.2%)	3 (10.0%)	
Coiling		33 (57.9%)	16 (59.3%)	17 (56.7%)	
Multiple		15 (26.3%)	5 (18.5%)	10 (33.3%)	
Rp Index (WU)	57	1.07 (0.91, 1.37)	1.05 (0.88, 1.5)	1.07 (0.92, 1.29)	0.74
Rs Index (WU)	57	10.2 (8.57, 12.1)	10.5 (9.01, 12.7)	10 (8.4, 11.8)	0.33
Rp/Rs ratio	57	0.11 (0.08, 0.14)	0.11 (0.08, 0.13)	0.11 (0.095, 0.14)	0.72
Transpulmonary gradient	57				0.8
1		3 (5.3%)	2 (7.4%)	1 (3.3%)	
2		3 (5.3%)	2 (7.4%)	1 (3.3%)	
3		15 (26.3%)	6 (22.2%)	9 (30.0%)	
4		19 (33.3%)	10 (37.0%)	9 (30.0%)	
5		12 (21.1%)	5 (18.5%)	7 (23.3%)	
6		3 (5.3%)	1 (3.7%)	2 (6.7%)	
9		1 (1.8%)	0 (0.0%)	1 (3.3%)	
11		1 (1.8%)	1 (3.7%)	0 (0.0%)	
Cath Contrast dose (mL)	47	63 (45, 76)	64 (44, 77)	58 (49.3, 75.5)	0.64
Cath Radiation (cGycm^2^)	47	166 (104, 218)	132 (89.5, 196)	186 (146, 274)	0.058
Cath time (hours)	47	2.93 (2.46, 3.48)	2.93 (2.36, 3.41)	2.94 (2.46, 3.74)	0.32

*SBP* systolic blood pressure

**Fig. 1:**
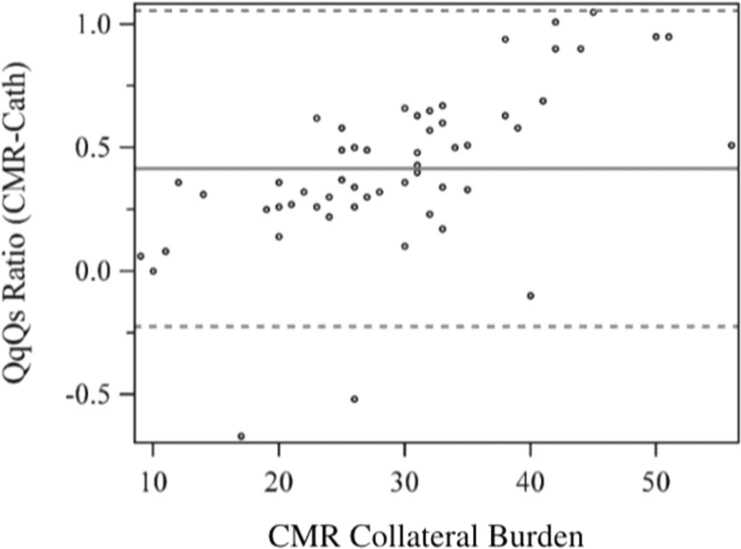
Bland-Altman plot for comparing CMR-Cath Qp/Qs difference with CMR-derived collateral burden. *CMR* cardiovascular magnetic resonance

**Fig. 2:**
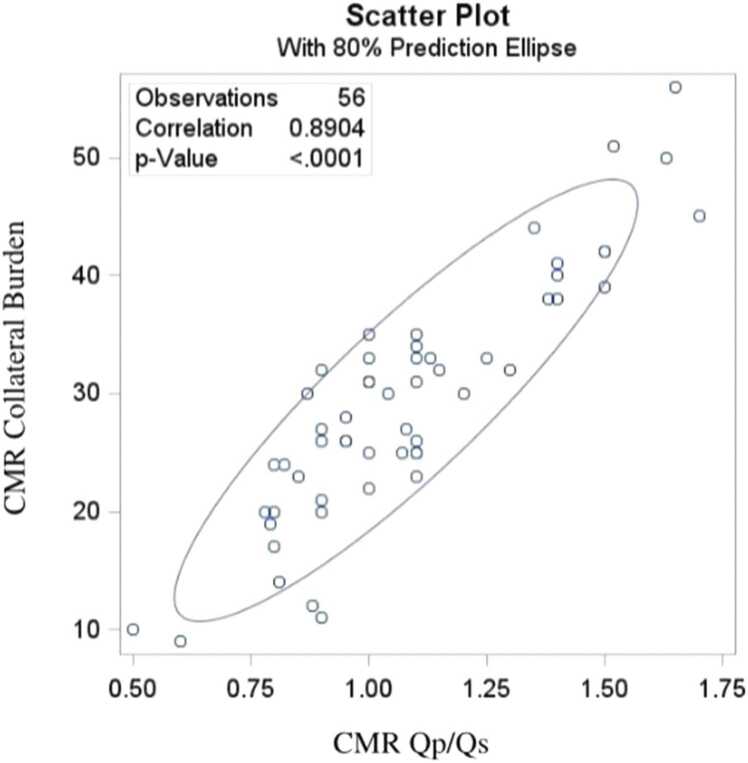
Scattered plot comparing CMR-derived collateral burden with CMR-derived Qp/Qs. *CMR* cardiovascular magnetic resonance imaging

**Fig. 3:**
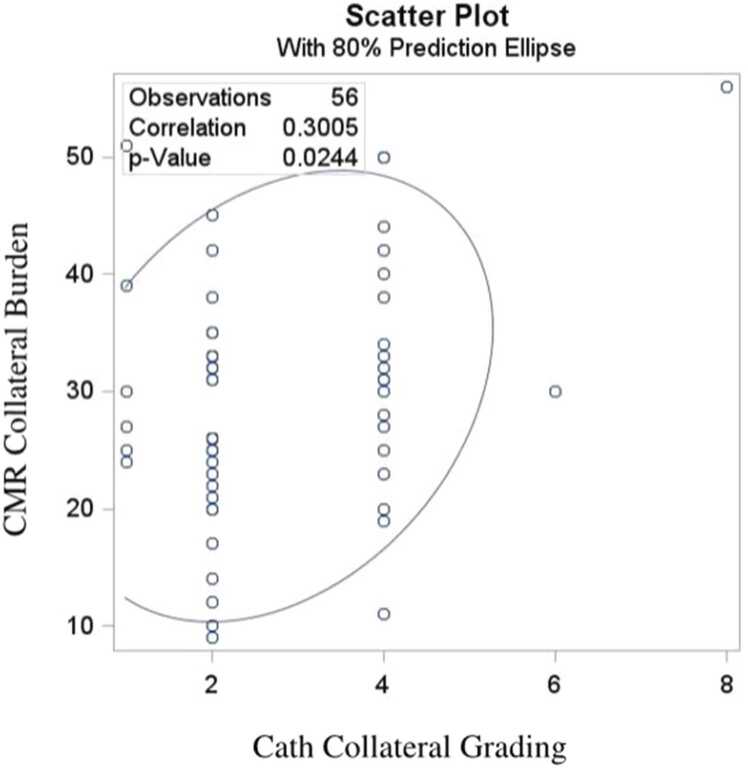
Scattered plot for correlating the CMR and Cath-derived collateral burden. *CMR* cardiovascular magnetic resonance imaging

Catheterization consistently underestimated Qp/Qs values, with a mean underestimation of 0.41 by Cath. This difference was strongly correlated with the burden of APC (p<0.0001) (Fig. 1). There was a strong correlation between CMR-derived Qp/Qs and APC burden (p<0.0001) (Fig. 2). The cath collateral grading did correlate with CMR collateral burden (p = 0.024) with extensive overlap (Fig. 3).

Postoperative outcomes, including chest tube duration and hospital length of stay, were not significantly affected by the presence of higher APC burden. While the higher APC group had a marginally longer median hospital stay (11.5 vs. 10 days), this difference lacked statistical significance. Among the cohort, 46 patients (80.7%) received catheter-based interventions, including APC occlusion using coils, devices, or microparticles, guided by angiographic findings. Notably, these targeted occlusions during cardiac Cath did not significantly alter post-surgical clinical variables.

**Fig. 4:**
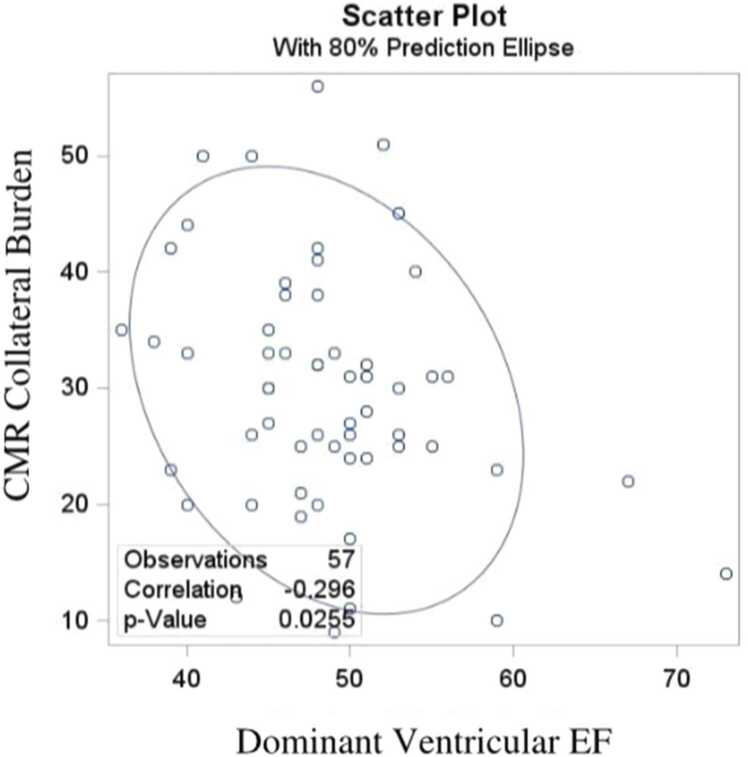
Scattered plot for correlating dominant ventricle EF with CMR-derived collateral burden. *CMR* cardiovascular magnetic resonance imaging, *EF* ejection fraction

## 4 Discussion

This study provides a comprehensive assessment of the combined use of CMR and Cath prior to Fontan completion, emphasizing the significant impact of APC burden on CMR-derived Qp/Qs, as well as on patient hemodynamics. Our findings highlight three key points as follows: (1) APC burden is common in the pre-Fontan population and is associated with adverse ventricular remodeling; (2) catheterization-derived Qp/Qs systematically underestimates pulmonary blood flow in the presence of significant collateralization; and (3) catheterization-based qualitative or semi-quantitative collateral assessment does not reliably discriminate patients with high collateral burden as defined by CMR.

APC burden has long been recognized as an important issue in single-ventricle patients prior to Fontan palliation. Glatz et al. identified several potential contributors to the formation of APCs, such as tissue hypoxia, cyanosis, inflammation, and diminished pulmonary blood flow, collectively implying that APC is a marker of overall unfavorable physiology [9]. Numerous studies have shown that the presence of APCs is associated with increased complications and poor outcomes, including chest tube duration and length of stay [7], [8], [9]. Our study similarly showed that over half of our pre-Fontan patients had a higher APC collateral burden, leading to higher Qp/Qs ratios than would traditionally be recognized by standard Cath assessment. Fittingly, this increased volume load from APCs leads to larger ventricular volumes, elevated end-diastolic pressures as well as worsening ventricular function. Our patient cohort with >50% having a collateral burden of >30% is in keeping with prior work from Grosse-Wortmann et al. and Prakash et al. where AP collateral median (interquartile range) values were 35% (11–62) and 29% (20%–35%), respectively [7], [10]. Previous studies have demonstrated over time Fontan patients demonstrated decreased collateral burden [13]. However, these older studies may be impacted by survival bias. Most recently, Latus et al. demonstrated serial assessment of Fontan patients and found no significant change in APC burden in patients with systemic right ventricles [14]. Our study did not find statistical clinical associations with APC burden or APC-related interventions, hospital length of stay and chest tube duration, which we believe is secondary to a relatively small patient population and low statistical power. Given other literature discussed above, we believe APC burden by CMR is an important metric to follow both prior to and after Fontan completion.

Our study also highlights the CMR’s added benefit to APC burden assessment and the relationship to Qp/Qs compared to the standard Cath-only approach. Hart et al. first noted the discrepancy between the CMR and Cath-based Qp/Qs related to APC burden, finding a mean difference of 0.36 in 10 pre-Fontan patients. We found a similar difference in our cohort of 57 pre-Fontan patients of 0.41 [15]. Given the limitations of traditional Cath oximetric methods, which are unable to sample distal to the APCs, our paper confirms that CMR and Cath measurements cannot be used interchangeably. An additional aspect of our paper highlights the challenge of standard angiography to assess relative collateral burden. Traditional methods to angiographically assess the severity of collaterals have been primarily subjective [10], [16]. Prakash et al. compared semi-quantitative angiographic grading and found significant overall agreement between grades and APC burden. Our study attempted to provide a more objective evaluation incorporating visual collateral presence and oxygen saturation step up into the pulmonary arteries [10]. Similar to Prakash et al., our 8-point grading scale did correlate with the APC burden, but with substantial overlap. These results demonstrate the importance of combining CMR and Cath pre-Fontan, given the importance of APC determination.

Addition potential benefits regarding combined CMR-Cath assessment in the pre-Fontan evaluation involve guidance toward coiling of collaterals. Goldstein et al. using data from the multicenter C3PO registry, found that aortopulmonary collateral occlusion was among the most frequently performed interventions during pre-Fontan Cath [17]. Eilers et al. demonstrated that combined CMR and Cath assessment led to fewer patients undergoing collateral occlusion using an APC burden cutoff of 30% when compared to Cath assessment alone [11]. In our institution, the decision to intervene on APCs was left to the discretion of the interventional cardiologist, and the vast majority (80.7%) of patients underwent collateral coiling. Our study was therefore unable to assess whether coiling collaterals changed postoperative clinical variables due to the low numbers of children who did not receive intervention.

## 5 Limitations

This study has some limitations. First, as a single-center retrospective analysis, the findings may not be generalizable to other institutions or broader patient populations. The sample size was modest, with only 57 patients included and 47 undergoing Fontan completion, which may limit the statistical power to detect differences in outcomes or rare complications. Selection bias is possible, particularly since patients with poor CMR image quality and those not undergoing both CMR and Cath under the same anesthesia were excluded, potentially omitting more complex or unstable cases.

Hemodynamic measurements obtained under general anesthesia may not fully reflect physiological conditions. There could be a potential variation in anesthetic support settings during the CMR and Cath procedures, potentially confounding the comparison between CMR and Cath-derived Qp/Qs values. The quantification of APC burden via CMR, while validated, can be influenced by technical factors such as image quality and operator experience and may not capture all relevant collateral flow in every patient.

The decision to intervene on APCs was left at the discretion of the interventional cardiologist, introducing variability in management. The study focused on immediate postoperative outcomes, with limited follow-up, so the long-term impact of APC burden and interventions remains unclear. Prospective, multicenter studies with standardized protocols and extended follow-up are needed to confirm these findings and clarify their implications for long-term patient outcomes.

Lastly, although the majority (>75%) of centers associated with the Single Ventricle Reconstruction Trial report performing cardiac cath on all patients prior to Fontan procedure, important questions have been brought up regarding a CMR-only approach for certain low-risk patients [18]. This work has previously been discussed by Fogel et al. where certain single-ventricle patients with reassuring findings on echocardiogram and CMR were shown to undergo successful Fontan completion with similar short-term outcomes to cardiac cath assessed patients [19]. Further work by Quail et al. has retrospectively reviewed CMR flows and internal jugular venous pressure assessment to estimate central venous pressure after Fontan completion [20]. Our institution's experience with combined CMR and Cath assessment was not designed to address that important question. Further work will be important to determine whether AP coiling at certain collateral burden percentages changes postoperative outcomes, and if aggressive AP coiling is not necessary whether the CMR alone pre-Fontan assessment has a high sensitivity at identifying the rare patients at risk for early Fontan failure.

## 6 Conclusion

In summary, this study emphasizes the value of integrating CMR with Cath for pre-Fontan evaluation in single-ventricle patients. Our findings confirm that CMR provides more accurate quantification of Qp/Qs and APC burden than Cath alone, especially in the presence of significant collateral flow. While the higher APC burden was associated with adverse preoperative hemodynamics, it did not correlate with worse immediate postoperative outcomes in our cohort, likely due to the small sample size and lower statistical power. These results also advocate for a tailored approach to APC intervention and highlight the evolving role of CMR as a noninvasive tool for risk stratification and surgical planning in this complex population. Further multicenter studies are warranted to clarify long-term implications.

## Author contributions

**Ashish Shrivastava:** Writing – review & editing, Writing – original draft, Methodology, Investigation, Formal analysis, Data curation, Conceptualization. **Russel Hirsch:** Supervision, Methodology. **Shabana Shahanavaz:** Validation, Supervision. **Cara E. Morin:** Writing – review & editing, Validation, Supervision, Methodology. **Todd Jenkins:** Validation, Formal analysis. **Zhiqian Gao:** Validation, Supervision, Formal analysis, Data curation. **Sean M. Lang:** Supervision, Methodology, Investigation, Formal analysis.

## Declaration of competing interests

The authors declare that they have no known competing financial interests or personal relationships that could have appeared to influence the work reported in this paper.
